# Notch1 Pathway Protects against Burn-Induced Myocardial Injury by Repressing Reactive Oxygen Species Production through JAK2/STAT3 Signaling

**DOI:** 10.1155/2016/5638943

**Published:** 2016-01-05

**Authors:** Weixia Cai, Xuekang Yang, Shichao Han, Haitao Guo, Zhao Zheng, Hongtao Wang, Hao Guan, Yanhui Jia, Jianxin Gao, Tao Yang, Xiongxiang Zhu, Dahai Hu

**Affiliations:** ^1^Department of Burns and Cutaneous Surgery, Xijing Hospital, Fourth Military Medical University, Xi'an, Shaanxi 710032, China; ^2^Department of Physiology, School of Basic Medical Sciences, Fourth Military Medical University, Xi'an, Shaanxi 710032, China; ^3^First Student Brigade, Fourth Military Medical University, Xi'an, Shaanxi 710032, China

## Abstract

Oxidative stress plays an important role in burn-induced myocardial injury, but the cellular mechanisms that control reactive oxygen species (ROS) production and scavenging are not fully understood. This study demonstrated that blockade of Notch signaling via knockout of the transcription factor RBP-J or a pharmacological inhibitor aggravated postburn myocardial injury, which manifested as deteriorated serum CK, CK-MB, and LDH levels and increased apoptosis* in vitro* and* in vivo*. Interruption of Notch signaling increased intracellular ROS production, and a ROS scavenger reversed the exacerbated myocardial injury after Notch signaling blockade. These results suggest that Notch signaling deficiency aggravated postburn myocardial injury through increased ROS levels. Notch signaling blockade also decreased MnSOD expression* in vitro* and* in vivo*. Notably, Notch signaling blockade downregulated p-JAK2 and p-STAT3 expression. Inhibition of JAK2/STAT3 signaling with AG490 markedly decreased MnSOD expression, increased ROS production, and aggravated myocardial injury. AG490 plus GSI exerted no additional effects. These results demonstrate that Notch signaling protects against burn-induced myocardial injury through JAK2/STAT3 signaling, which activates the expression of MnSOD and leads to decreased ROS levels.

## 1. Introduction

Severe burn injury results in multiple organ dysfunction, which is the leading cause of death in intensive care units (ICUs) [1, 2]. Myocardial injury is a major contributor to mortality, particularly in individuals with preexisting cardiac pathology [3, 4]. Numerous experimental studies have investigated the molecular mechanisms involved in burn-induced myocardial injury to create novel therapeutic interventions and agents to reduce the incidence of life-threatening complications. However, there is still a lack of effective therapies that increase myocardial resistance to burn injury despite decades of laboratory studies and clinical practice.

Mounting evidence demonstrates that oxidative stress plays an important role in burn-induced myocardial injury [5–7]. However, the cellular mechanisms that control ROS production and scavenging are not fully understood. The Notch pathway is an evolutionarily conserved signaling system that plays a crucial role in cell fate decisions, differentiation, proliferation, and apoptosis. Four Notch receptors (Notch1–4) and five Notch ligands (Delta-like1, 3, 4, and Jagged1, 2) have been identified in mammals. The binding of a Notch ligand to its receptor triggers *γ*-secretase-mediated proteolytic cleavage of the Notch intracellular domain (NICD), which translocates to the nucleus to form a transcription-activating complex. This complex mediates the transcription of downstream target genes such as Hes1, Hey1, and cyclin D [8].

Jagged1 and Notch1 are expressed in the adult heart [9], and these proteins protect cardiac tissue under various pathophysiological conditions [10], including alcoholic cardiomyopathy, myocardial infarction [11], cardiac hypertrophy [12], and ischemia-reperfusion injury [13]. More recent studies have revealed that Notch1 suppresses oxidative stress in hepatocytes [14] and endothelial cells [15]. Notably, we recently found that Notch1 protected against MI/R injury via the reduction of oxidative/nitrative stress [13]. However, whether Notch1 signaling plays a role in burn-induced myocardial injury has yet to be determined. This study demonstrated that the Notch1 pathway protected against postburn myocardial injury via the repression of reactive oxygen species (ROS) production through JAK2/STAT3 signaling.

## 2. Materials and Methods

### 2.1. Animals

Healthy adult male Sprague-Dawley (SD) rats (weighing 200~250 g) or newborn SD rats (1–3 days old) were obtained from the Experimental Animal Center of Fourth Military Medical University. All animal experiments were performed in accordance with the guidelines from the Administration of Animal Experiments for Medical Research Purposes issued by the Ministry of Health of China. The animal ethics number was XJYYLL-2014177. Animals were fed ad libitum standard diet and water throughout the study. All animals were housed separately and kept under standard conditions at room temperature (22~24°C) in a 12 h light/12 h dark cycle.

Conditional RBP-J allele (RBP-J floxed) mice were a generous gift from Professor Hua Han, M.D., Ph.D. (Department of Medical Genetics and Developmental Biology, Fourth Military Medical University). Myh6-Cre mice were obtained from Model Animal Research Center of Nanjing University. Cardio-specific RBP-J knockout mice were generated from RBP-J floxed mice and Myh6-Cre mice. Tamoxifen was administered to each mouse, when mouse reached 6 weeks after birth, at 50 mg/kg by intraperitoneal injections once a day for 5 days.

### 2.2. Burn Procedure

Animals were anesthetized lightly with pentobarbital sodium (50 mg/kg). Rats were placed in a prefabricated template with a rectangular opening that exposed the dorsal skin surface but protected the remaining skin from burn exposure. This template limits the burn area to a predetermined 30% TBSA. The exposed skin surface was immersed in 95°C water for 18 s as previously described [16, 17]. Sham-burn control animals were treated identically to animals in the burn group, except that the skin was immersed in 37°C water. Mice (18–22 g) were then placed in a template estimating 30% total body surface area and subjected to a steam burn for 8 s as previously described [18]. Animals were immediately infused with Ringer's lactate solution according to Parkland's formula and received a subcutaneous injection of normal saline with 0.1 mg/kg of buprenorphine (Sigma, St. Louis, MO) for pain control. Samples were collected from burn and sham animals after the burn procedure at various time points.

### 2.3. Serum Collection

Animals were killed under anesthesia at each endpoint after burn injury. Rats blood samples were taken from the aortaventralis. Mice blood samples were taken by eyeball extirpating. The collected blood was centrifuged at 1500 g for 10 minutes, and the serum was gathered and stored at −80°C for further use. Serums from rats 12 h after burn were used to challenge cardiomyocytes.

### 2.4. Cardiomyocyte Culture and Stimulation with Burn Serum

Neonatal rat cardiomyocytes were isolated from the ventricles of one to 3-day-old Sprague-Dawley rats using a previously described procedure [19]. Cells enriched for cardiomyocytes were placed on 6 plates and maintained in Cardiac Myocyte Medium (CMM, ScienCell, USA), which included cardiac myocyte growth supplement (CMGS) and a low concentration of fetal bovine serum (5%) in a CO_2_ incubator at 37°C at the indicated time points. A *γ*-secretase inhibitor (GSI; Calbiochem, La Jolla, CA) was used at the concentration of 75 *μ*M, with dimethyl sulfoxide (DMSO) as a control. Burn serum was added at 15% (v/v).

### 2.5. Evaluation of Myocardial Injury and Apoptosis

Myocardial injury was evaluated using mice serum creatine kinase (CK), the MB isoenzyme of creatine kinase (CK-MB), and lactate dehydrogenase (LDH). All assays were performed using a chemistry autoanalyzer (Vitros 750, Johnson & Johnson, Rochester, USA). Cardiomyocyte injury was assessed via measurement of LDH release into the culture medium. Briefly, the incubation medium was stored at 4°C at the end of treatment, and the same volume of cold buffer (10 mM Tris–HCl [pH 7.4], 1 mM EDTA) was added to the cells. The cells were scraped and lysed using trituration. Lysates were centrifuged at 4°C, and the supernatant was stored at 4°C. LDH levels in the medium (released LDH) and cell lysate (retained LDH) were measured using a spectrophotometric assay. The results are expressed as the percent released LDH compared with total (released plus retained) LDH.

Myocardial apoptosis was analyzed using TUNEL assays and an in situ cell death detection kit according to the manufacturers' protocol. Cardiomyocyte apoptosis was evaluated using acridine orange (AO)/ethidium bromide (EB). Cells were harvested at 70–80% confluency, seeded in 48-well plates at 2 × 10^5^ cells/well, and incubated overnight for the cells to attach. The AO/EB solution was prepared with 100 *μ*g/mL of each reagent. Cells were treated for 12 h with Burn serum, and each sample was stained with 100 *μ*L of AO/EB solution just prior to microscopy and quantification. At least 300 cells were counted for each treatment, and the percentages of apoptotic (red-orange nucleus) and live (green nucleus) cells were calculated.

### 2.6. ROS Measurement

Cardiomyocytes were labeled with 2′, 7′-dichlorofluorescein (DCFH-DA) (S0033, Bryotime, Shanghai, China) for the measurement of intracellular ROS generation following recommended protocols. Intracellular ROS levels were determined via measurement of the oxidative conversion of cell-permeable DCFH-DA to fluorescent dichlorofluorescein (DCF). Cells were incubated with DCFH-DA at 37°C for 20 min. The DCF fluorescence distribution of 200,000 cells was detected using flow cytometry at an excitation wavelength of 488 nm and an emission wavelength of 535 nm. The levels of intracellular ROS were quantified using mean fluorescent intensity (MFI), and the results were statistically compared between groups, as described.

Myocardial superoxide anion content was determined using lucigenin-enhanced luminescence [13]. Samples were weighed, cut into uniform cubes (0.5 mm^3^), and transferred into polypropylene tubes containing 1 mL of PBS and lucigenin (Sigma, 0.25 mmol/L). Tubes were placed in an FB12-Berthold luminometer (Berthold Technologies, Bad Wildbad, Germany). The RLU emitted was recorded and integrated over 30 s intervals for 5 min. Activity was normalized to dry tissue weights.

### 2.7. Western Blot Analysis

Myocardial and cardiomyocyte samples were homogenized in lysis buffer containing 20 mM Tris-HCl (pH 7.4), 150 mM NaCl, 5 mM EDTA, 1% Triton-X 100, 1% DTT, and 1% of a protease inhibitor cocktail. Lysates were centrifuged at 12,000 ×g for 15 min, and the resulting supernatants were transferred to a new tube and stored at −70°C. Protein concentrations were determined using a Bradford protein assay kit. Equal amounts of proteins were separated using 10% sodium dodecyl sulfate-polyacrylamide gel electrophoresis (SDS-PAGE) and transferred onto nitrocellulose membranes. Membranes were blocked for 1 h in Tris-buffered saline and Tween 20 (TBST, pH 7.6) containing 5% nonfat dry milk and incubated overnight at 4°C with antibodies against Notch1 ICD and Hes1 (Abcam, Cambridge, MA), MnSOD (Santa Cruz Biotechnology), JAK2/phospho-JAK2 (Abcam, Cambridge, MA), STAT3/phospho-STAT3, and GAPDH (Cell Signaling Technology, Danvers, MA), followed by washes with TBST. The membranes were probed with appropriate secondary antibodies (1 : 3000 dilution) at room temperature for 90 min and washed with TBST. Protein bands were detected using a Bio-Rad imaging system (Bio-Rad, Hercules, CA, USA) and quantified using the Quantity One software package (West Berkeley, CA, USA).

### 2.8. Statistical Analysis

All values in the text and figures are presented as means ± SEM. Data (except for Western blot density) were subjected to ANOVA followed by the Bonferroni correction for post hoc *t*-tests. Western blot densities were analyzed using the Kruskal-Wallis test followed by Dunn's post hoc test. *p* values < 0.05 were considered statistically significant.

## 3. Results

### 3.1. Notch1 Pathway Responds to Myocardial Injury after Burn

**Figure 1:**
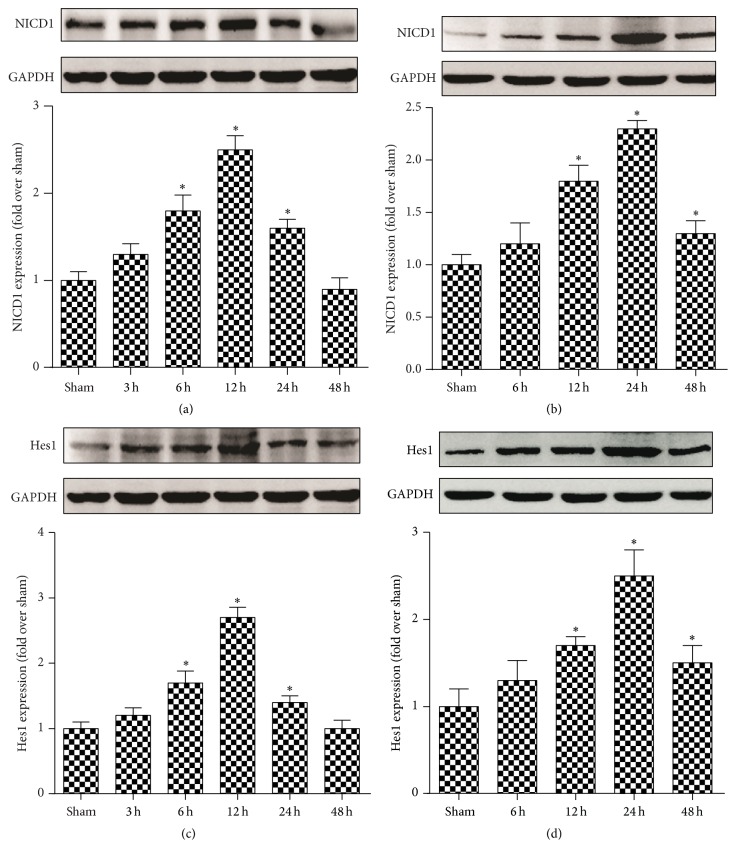
Notch1 pathway responds to myocardial injury after burn. Mice were subjected to burn injury. (a, c) Protein expression of Notch1 intracellular domain (NICD) and Hes1 in mouse myocardial tissue after burn injury over time. Rat cardiomyocytes were challenged with burn serum* in vitro*. (b, d) showed the NICD1 and Hes1 protein expression in cardiomyocytes. The values presented are the mean ± SEM (*n* = 8 per group). ^*∗*^ *p* < 0.05 compared to the value in sham groups.

Normal mice were subjected to burn injury, and the protein expression of Notch1 and Hes1 in myocardium tissue was examined at various time points from 0 to 24 h. Notch1 ICD protein levels, which are a marker of Notch1 activation, were significantly increased at an early time point (3 and 6 h) after burn application. The maximum Notch1 ICD protein levels occurred 12 h after burn application and decreased at 24 and 48 h (Figure 1(a)). Protein levels of Hes1, which is a downstream effector of Notch1 signaling, reached their peak at 12 h and were downregulated at 24 and 48 h (Figure 1(c)). A similar trend was observed in cultured rat cardiomyocytes after exposure to burn serum (Figures 1(b) and 1(d)). Notch1 ICD and Hes1 protein expression increased at early time points (6, 12 , and 24 h) and decreased at a later time point (48 h). These results demonstrated that cardiac Notch1 signaling was activated during burn injury.

### 3.2. Notch Signal Blockade Aggravates Burn-Induced Cardiomyocyte Apoptosis* In Vitro*

**Figure 2:**
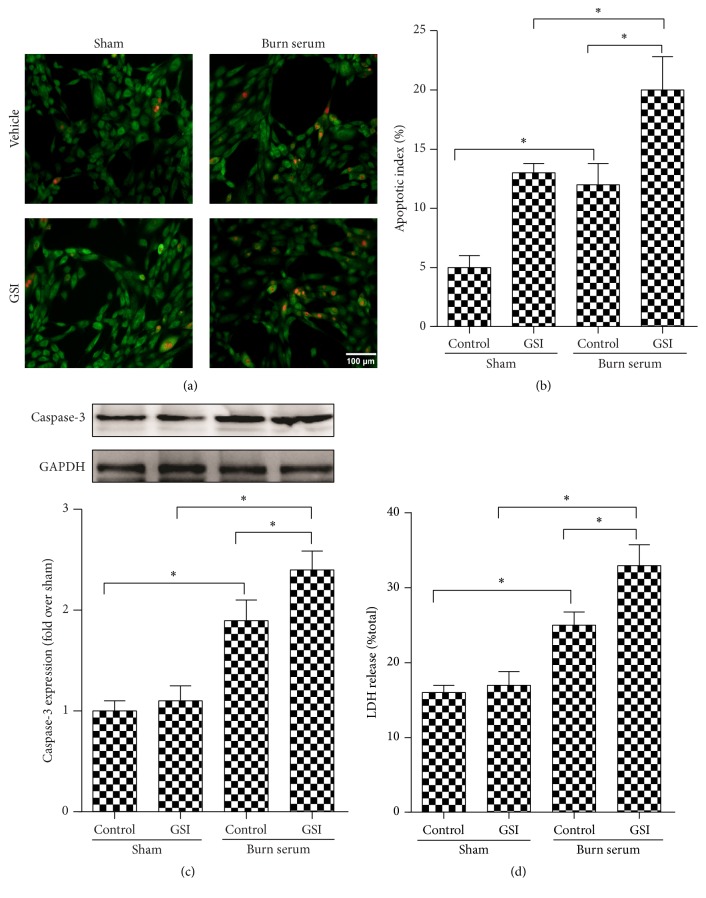
Notch signal blockade aggravates burn-induced cardiomyocyte apoptosis* in vitro*. Rat cardiomyocytes were challenged with burn serum* in vitro* in the presence of DMSO or GSI. Apoptotic cells were stained by AO/EB staining 12 hours after challenge (a, magnification ×200) and were quantified (b). Cells were collected to determine the expression of caspase-3 protein expression (c). Cell supernatants in (c) were collected and LDH production (d) was assessed. The values presented are the mean ± SEM (*n* = 8 per group). ^*∗*^ *p* < 0.05.

Rat cardiomyocytes were pretreated with GSI or vehicle, followed by the addition of burn serum to investigate the role of Notch1 signaling in cardiomyocytes postburn injury. AO/EB staining revealed significantly elevated apoptosis in cells challenged with burn serum. Notably, burn serum induced remarkably increased apoptosis when Notch signaling was blocked by GSI (Figures 2(a) and 2(b)). Burn serum also caused a significant increase in caspase-3 expression, and blockade of Notch1 signaling by GSI further aggravated caspase-3 expression (Figure 2(c)). GSI also significantly aggravated cardiomyocyte injury, as evidenced by increased LDH levels in the supernatants (Figure 2(d)).

### 3.3. Notch Signal Deficiency Exacerbates Burn-Induced Myocardial Injury* In Vivo*

**Figure 3:**
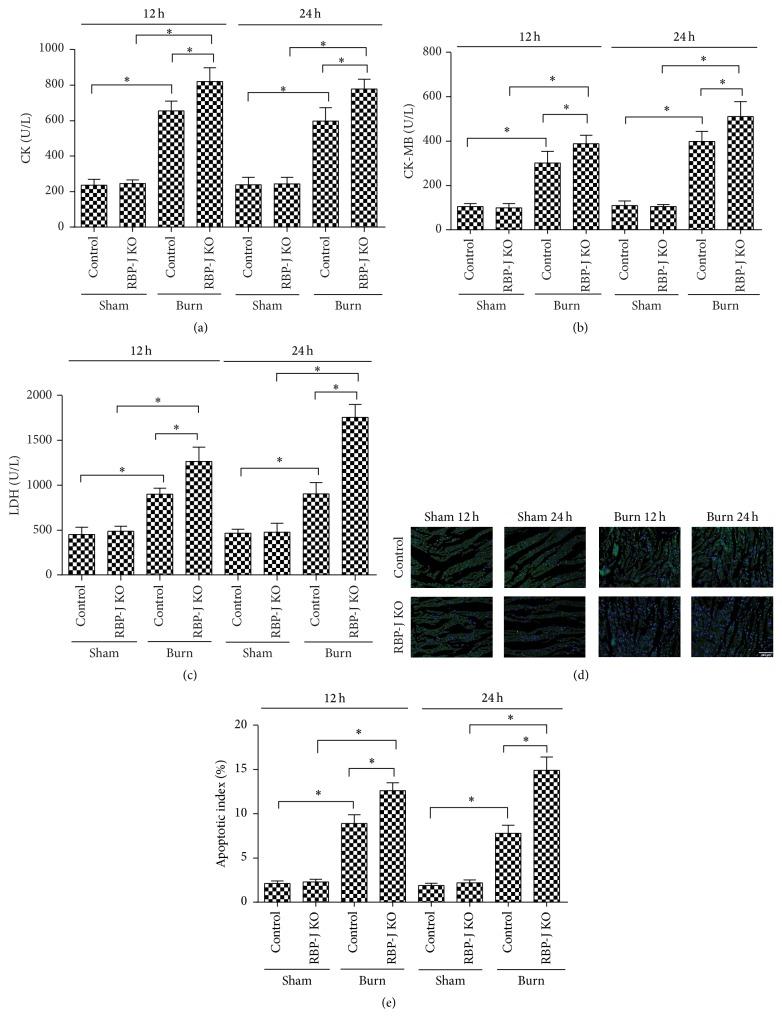
Notch signal deficiency exacerbates burn-induced myocardial injury* in vivo*. RBP-J KO or control mice were subjected to burn injury and were examined 12 hours or 24 hours after injury. Serum CK (a), CK-MB (b), and LDH (c). (d) Apoptotic cells were stained by TUNEL staining in myocardial tissues. (e) Quantitative comparison of apoptotic cells upon TUNEL staining in (d). The values presented are the mean ± SEM (*n* = 8 per group). ^*∗*^ *p* < 0.05.

We used a conditional RBP-J-knockout approach to further elucidate the role of the Notch1 signaling pathway in the postburn myocardium. Significantly higher levels of serum CK, CK-MB, and LDH were detected in RBP-J KO mice at 12 h and 24 h postburn injury compared to normal mice subjected to burn injury (Figures 3(a)–3(c)). TUNEL staining revealed that burn injury induced more apoptotic cells in RBP-J KO mice than in normal mice (Figures 3(d) and 3(e)). These data suggest that disruption of Notch1 signaling aggravated postburn myocardial injury.

### 3.4. Notch Blockade Leads to Increased ROS Production

**Figure 4:**
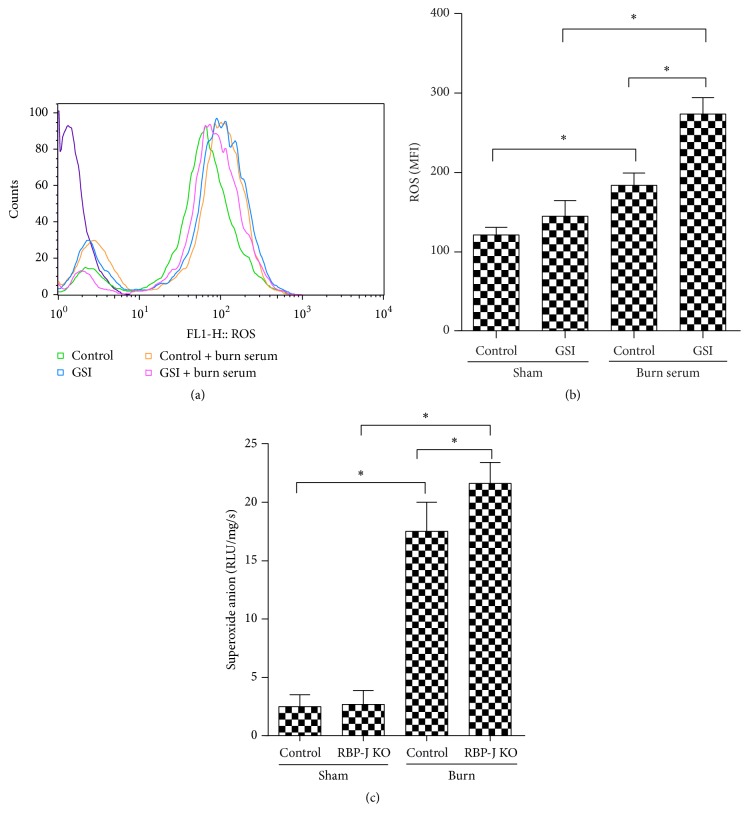
Notch blockade leads to increased ROS production. Rat cardiomyocytes were challenged with burn serum* in vitro* in the presence of DMSO or GSI. ROS were examined by way of FACS (a) and were quantified by way of mean fluorescence intensity (b). RBP-J KO or control mice were subjected to burn injury and were examined 12 hours after injury. (c) Myocardium tissues were isolated and examined for superoxide anions content by way of lucigenin-enhanced luminescence. The values presented are the mean ± SEM (*n* = 8 per group). ^*∗*^ *p* < 0.05.

**Figure 5:**
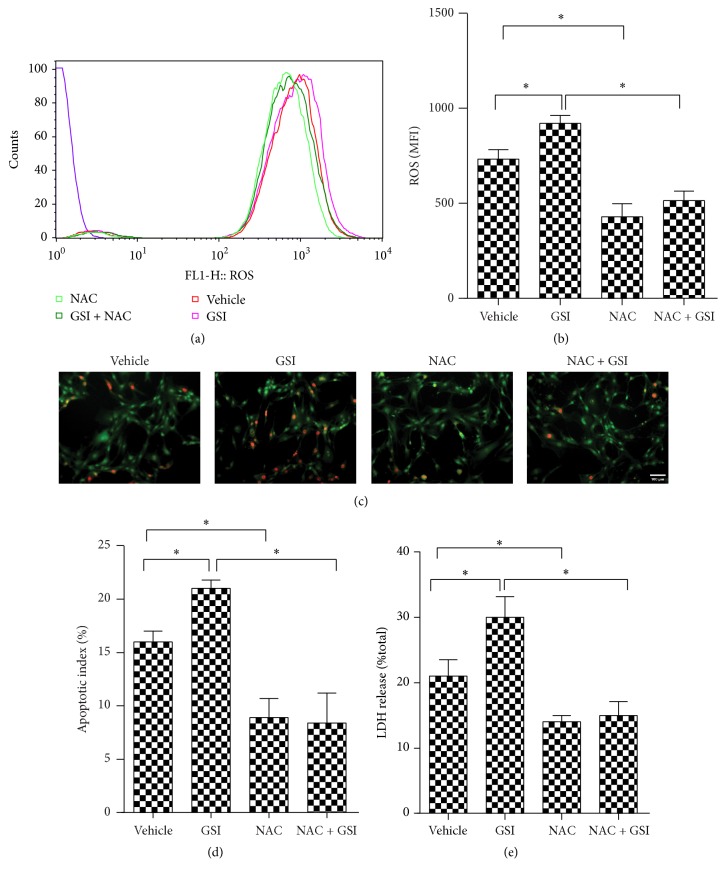
ROS scavenger alleviates burn-induced myocardial injury aggravated by Notch signal deficiency. Rat cardiomyocytes were challenged with burn serum* in vitro* in the presence of DMSO or GSI, with or without the ROS scavenger, NAC. ROS were examined by way of FACS (a) and were quantified by way of mean fluorescence intensity (b). (c) Apoptotic cells were stained by AO/EB staining. (d) Quantitative comparison of apoptotic cells upon AO/EB staining in (c). Cell supernatants were collected and LDH production (e) was assessed. The values presented are the mean ± SEM (*n* = 8 per group). ^*∗*^ *p* < 0.05.

We examined ROS production in rat cardiomyocytes treated with burn serum in the absence of Notch signaling. Figures 4(a) and 4(b) show that burn serum markedly increased ROS levels in cardiomyocytes as determined by FACS, and blockade of Notch signaling with GSI remarkably increased ROS levels after burn serum challenge. In the* in vivo* experiments, mice were subjected to burn injury for 12 h. The same phenomena were detected in RBP-J KO mice (Figure 4(c)). Superoxide production in the RBP-J KO mice was significantly increased in KO mice compared to control mice. These results suggested that the exacerbated myocardial injury resulting from Notch blockade was mediated by an increase in ROS production. We pretreated cardiomyocytes with GSI and exposed the cells to burn serum, followed by treatment with the ROS scavenger NAC to further investigate this hypothesis. GSI remarkably increased ROS levels in rat cardiomyocytes after burn serum challenge. In sharp contrast, NAC effectively decreased ROS in the GSI-treated and vehicle groups (Figures 5(a) and 5(b)). The GSI-treated group exhibited aggravated apoptosis and LDH levels compared to the vehicle group. Notably, NAC significantly reduced cardiomyocyte apoptosis and LDH levels (Figures 5(c), 5(d), and 5(e)). These findings suggest that blockade of Notch signaling aggravated postburn myocardial injury through increased ROS production.

### 3.5. Disruption of Notch Signal Leads to Downregulation of MnSOD

**Figure 6:**
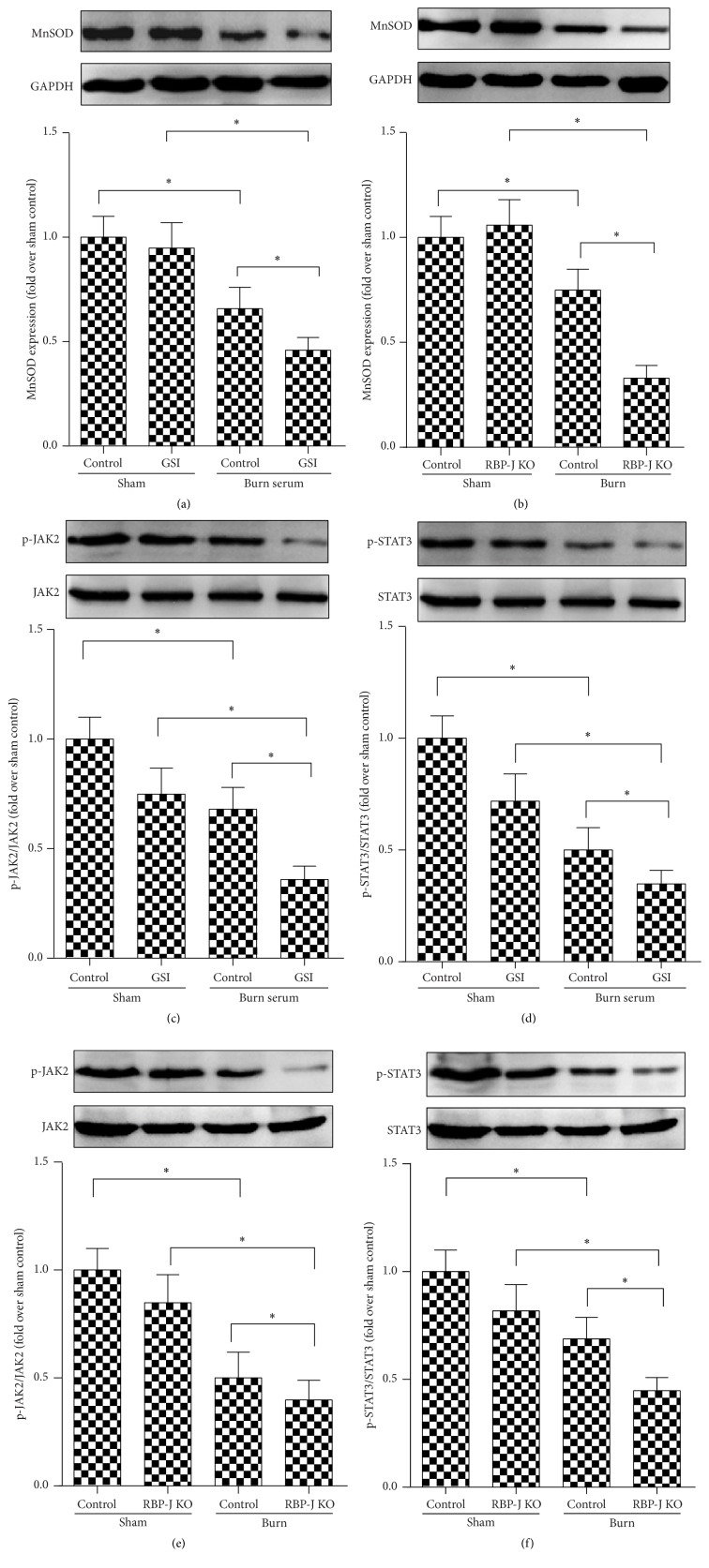
Notch signal blockade attenuates MnSOD and JAK2/STAT3 activation during burn injury. Rat cardiomyocytes were challenged with burn serum* in vitro* in the presence of DMSO or GSI. MnSOD (a) and JAK2/STAT3 (c, d) expression was determined by western blot in cardiomyocytes. MnSOD (b) and JAK2/STAT3 (e, f) expression in myocardial tissues of RBP-JKO and control mice subjected to burn injury for 12 hours. The values presented are the mean ± SEM (*n* = 8 per group). ^*∗*^ *p* < 0.05.

Mitochondrial respiration provides more than 90% of intracellular ROS, which is scavenged by MnSOD. The expression of MnSOD in cardiomyocytes treated with burn serum in the presence of GSI was downregulated significantly (Figure 6(a)). In the* in vivo* experiments, RBP-J KO mice subjected to burn injury also exhibited a significant downregulation of MnSOD expression in the myocardium (Figure 6(b)). These data suggest that blockade of Notch signaling downregulated MnSOD expression, which increased ROS scavenging and aggravated myocardial injury.

### 3.6. Notch Signal Blockade Attenuates STAT3 Activation during Burn Injury

JAK2/STAT3 signaling transactivates MnSOD, which suggests that the inhibition of Notch1 signaling downregulates the transcription of MnSOD through decreased STAT3 activation and leads to increased ROS and aggravated oxidative stress injury. Figures 6(c) and 6(d) show that burn serum significantly decreased p-JAK2 and p-STAT3 expression, and the inhibition of Notch1 signaling by GSI further attenuated p-JAK2 and p-STAT3 expression. RBP-J KO mice subjected to burn injury also exhibited a remarkable further downregulation of p-JAK2 and p-STAT3 expression in the myocardium (Figures 6(e) and 6(f)).

### 3.7. Inhibition of JAK2/STAT3 Signaling Preferentially Aggravated Myocardial Injury after Burn

**Figure 7:**
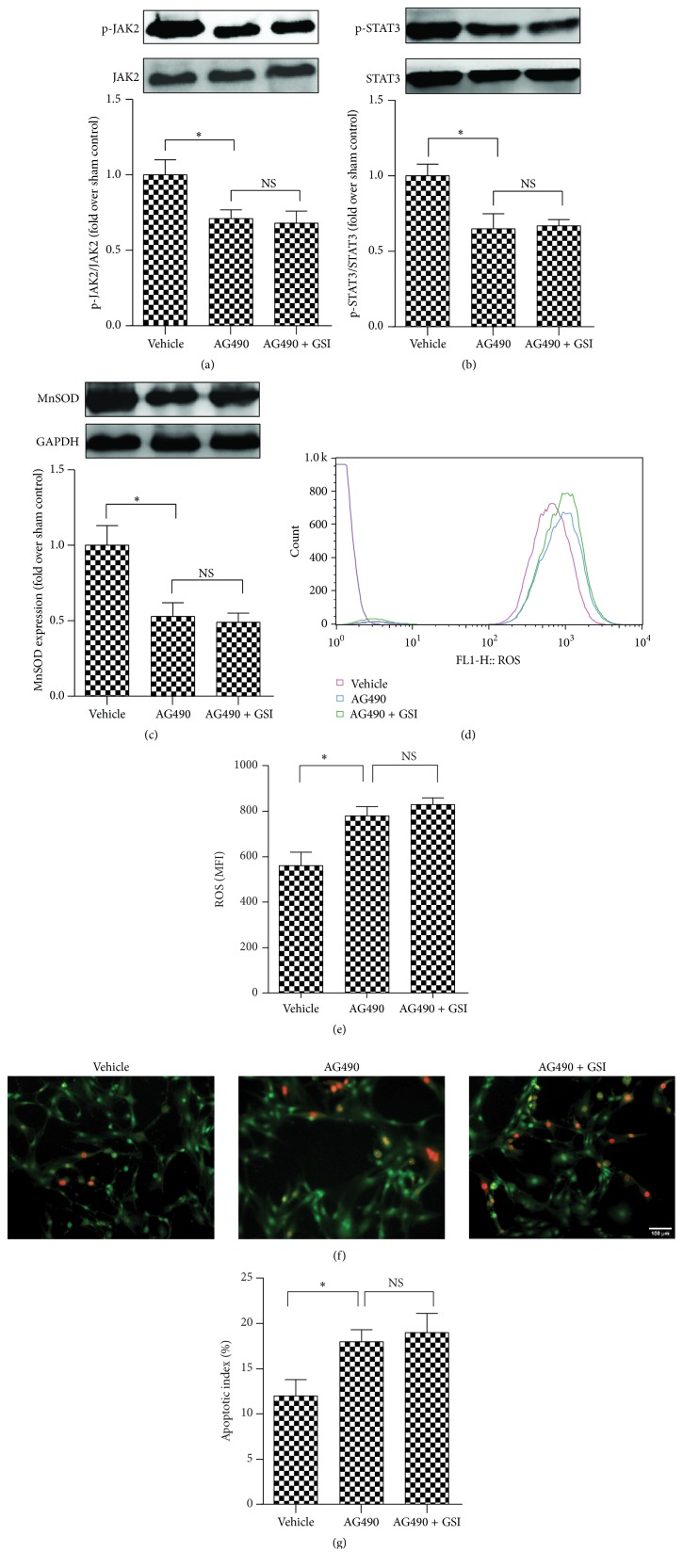
Inhibition of JAK2/STAT3 signaling preferentially aggravated myocardial injury after burn. Rat cardiomyocytes were challenged with burn serum* in vitro* in the presence of AG490, with or without GSI. JAK2/STAT3 (a, b) and MnSOD (c) expression was determined by western blot in cardiomyocytes. ROS were examined by way of FACS (d) and were quantified by way of mean fluorescence intensity (e). (f) Apoptotic cells were stained by AO/EB staining. (g) Quantitative comparison of apoptotic cells upon AO/EB staining in (f). The values presented are the mean ± SEM (*n* = 8 per group). ^*∗*^ *p* < 0.05. NS, not significant.

Our results suggest that the inhibition of Notch1 aggravates ROS production and myocardial injury via the inhibition of JAK2/STAT3 signaling. An additional series of experiments was performed to obtain more evidence to support this hypothesis. Rat cardiomyocytes were subjected to burn serum as described above and treated with either AG490 (a JAK2/STAT3 inhibitor, 2 *μ*M) or AG490 plus GSI. Figures 7(a) and 7(b) show that treatment with AG490 significantly reduced p-JAK2 and p-STAT3 expression. Treatment with GSI plus AG490 induced no additional effects. The expression of MnSOD had a consistent trend (Figure 7(c)). AG490 treatment also markedly increased oxidative stress and aggravated myocardial injury. Treatment with GSI plus AG490 induced no additional cardioprotective effects (Figures 7(d)–7(g)). These results demonstrated that the inhibition of JAK2/STAT3 signaling preferentially aggravated postburn myocardial injury, and JAK2/STAT3 signaling played a critical role in the cardioprotection of Notch1.

## 4. Discussion

This study produced the following major findings. First, genetic knockout or pharmacological inhibition of Notch1 significantly aggravated myocardial injury* in vitro* and* in vivo*. Second, we demonstrated for the first time that Notch signal blockade aggravated postburn myocardial injury via JAK2/STAT3 signaling, which downregulated MnSOD expression and increased ROS levels. These results indicate that endogenous Notch1 signaling is critical for burn-induced myocardial injury, and this signaling pathway may serve as a new therapeutic target.

Notch signaling is highly relevant for proper myocardial function and response to injury. Our previous study found that Notch1 knockdown significantly aggravated MI/R injury, as evidenced by an enlarged infarct size, depressed cardiac function, and increased myocardial apoptosis [13]. Activation of Notch1 by Jagged1 also attenuated MI/R injury. The present study examined the role of the Notch1 pathway in burn-induced myocardial injury for the first time and further confirmed the cardioprotective effects of the Notch1 pathway. These data are similar to the previous study in other experiments in which Notch signaling exerts protection in several pathophysiological conditions, including alcoholic cardiomyopathy [10], myocardial infarction [11], and cardiac hypertrophy [12].

ROS causes oxidative stress and acts as the major mediator of postburn myocardial injury [7, 20, 21]. Mounting evidence has demonstrated that the Notch1 pathway plays a key role in ROS production [22–24]. Our previous study also demonstrated that activation of Notch1 signaling inhibited ROS production in hepatic ischemia/reperfusion (I/R) injury and MI/R injury [14]. Our present data demonstrated that complete Notch deficiency increased burn-induced ROS production in Notch RBP-J knockout mice, which suggests that the cardioprotective effects of the Notch1 pathway are involved in the regulation of ROS.

Oxidative stress is an imbalance between the production and elimination of ROS [25]. Under normal condition, the body has a potent antioxidant defense system, fighting against excessive generation of ROS. However, during some pathological conditions, these natural antioxidant defenses are damaged or the excessive ROS is generated, and oxidative stress occurs, leading to structural and functional injury [26]. The antioxidant defense system in living organism is complex. Among them, MnSOD is the most crucial enzyme in the cellular antioxidant system [27].

In the present study, our results reported here indicate that MnSOD, which is a critical ROS scavenger in mitochondria, was markedly downregulated after Notch deficiency. These observations suggest that a reduction in SOD activity is responsible for the suppression of ROS afforded by Notch deficiency in the postburn myocardium.

The Janus kinase/signal transducer and activator of transcription (JAK/STAT) signaling pathway controls multiple biological processes in metazoan development and tissue homoeostasis [28]. Four mammalian JAKs (JAK1, 2, 3, and Tyk2) and seven mammalian STATs (STAT1, 2, 3, 4, 5a, 5b, and 6) have been identified [29]. The JAK2/STAT3 signaling pathway is a highly evolutionarily conserved pathway that is involved in cell proliferation, differentiation, apoptosis, and inflammation [30–32]. Recent studies found that the JAK2/STAT3 signaling pathway plays an important role in myocardial I/R injury [33–35]. Certain cardioprotective agents, including hydrogen sulfide and fasudil, protect the myocardium against I/R injury via activation of the JAK2/STAT3 survival pathway [36, 37]. Mounting evidence has confirmed that the JAK2/STAT3 signaling plays a critical role in the regulation of oxidative stress responses [35, 38]. Similarly, our present study found that inhibition of JAK2/STAT3 signaling by AG490 markedly increased oxidative stress and aggravated postburn myocardial injury, which supports the critical role of JAK2/STAT3 signaling in burn-induced myocardial injury.

JAK2/STAT3 signaling regulates the transcription of MnSOD [14]. Our present data demonstrated that blockade of Notch signaling markedly attenuated p-JAK2 and p-STAT3 expression, which suggests that the inhibition of Notch1 signaling downregulates MnSOD transcription via decreased STAT3 activation and leads to increased ROS and aggravated I/R injury. Inhibition of JAK2/STAT3 signaling also preferentially aggravated oxidative stress and postburn myocardial injury, and pretreatment with GSI had no effect. These results further support the notion that Notch signaling regulates oxidative stress and postburn myocardial injury via JAK2/STAT3 signaling. These data are similar to our previous study that demonstrated that canonical Notch signaling protects hepatocytes from I/R injury via the activation of JAK2/STAT3 signaling, which activates the expression of MnSOD and leads to ROS scavenging.

However, some limitations in this study need to be noted. The mechanism by which ROS is influenced after downregulation of Notch1 is quite complex. The Notch1 pathway has been reported to enhance Akt activity in myocardium [39]. Results from our previous study and others showed that PI3K/Akt signaling played a critical role in burn-induced cardiomyocyte apoptosis [40–42]. Interestingly, several studies indicated that Akt signaling regulated ROS production in several models [43–45]. These facts suggest that PI3K-Akt signaling may play an important role in the regulation of oxidative stress by Notch1 during burn injury. Further study is needed to further investigate the possible mechanism of oxidative stress by Notch1.

In conclusion, we used genetic knockout and pharmacological inhibition of Notch1 to demonstrate novel roles of Notch1 signaling in burn-induced myocardial injury. Notch signaling also protects against burn-induced myocardial injury through JAK2/STAT3 signaling, which activates MnSOD expression and inhibits gp91phox expression and leads to decreased ROS levels. These findings suggest new therapeutic targets to limit burn-associated myocardial injury.
